# Toward consistent reporting of sample characteristics in studies investigating the biological mechanisms of romantic love

**DOI:** 10.3389/fpsyg.2023.983419

**Published:** 2023-05-04

**Authors:** Adam Bode, Marta Kowal

**Affiliations:** ^1^School of Archaeology and Anthropology, ANU College of Arts and Social Sciences, The Australian National University, Canberra, ACT, Australia; ^2^IDN Being Human Lab, University of Wrocław, Wrocław, Poland

**Keywords:** romantic love, neuroimaging, endocrinology, genetics, methods, sample

## Abstract

In this non-systematic review, we consider the sample reporting practices of 42 studies up to and including 2021 investigating the biological mechanisms of romantic love (i.e., 31 neuroimaging studies, nine endocrinological studies, one genetics study, and one combined neuroimaging and genetics study). We searched scientific databases using key terms and drew on our and other authors’ knowledge to identify studies that investigated the mechanisms associated with romantic love using neuroimaging, endocrinological, and genetic methods. Only studies with a group or entire sample experiencing romantic love were included. The aim was to collate all relevant studies and determine the comparability of studies and ability to assess the generalizability of findings. We summarize how these studies report sex/gender, age, romantic love, relationship duration/time in love, and sample descriptors. We then outline the case for promoting comparability and the ability to determine generalizability in future studies. The findings indicate a limited ability to compare studies’ samples or make an assessment of the generalizability of findings. Existing studies are not representative of the general population in a particular country or globally. We conclude by presenting ideas about how best to report sex, age, romantic love characteristics, relationship status, time in love, relationship duration, relationship satisfaction, type of unrequited love, sexual activity, cultural characteristics, socio-economic status, student status, and method-relevant descriptors. If our ideas are adopted, in part or in whole, we expect the comparability of studies to increase. Adopting our ideas will also make it easier to make an assessment of the generalizability of findings.

## 1. Introduction

Over the past two decades, scientists have developed a rudimentary understanding of the biological mechanisms that contribute to romantic love. These mechanisms include the neurobiology, endocrinology, and genetics of romantic love. However, studies that tackled these issues have a significant limitation–the samples’ characteristics impact their findings. Age, sex, demographic characteristics, relationship characteristics, and romantic love characteristics are but a few features that could affect the comparability of studies and generalizability (Kukull and Ganguli, 2012) of findings. While the findings of neuroimaging studies are generally coherent (see Bode and Kushnick, 2021), the inconsistent endocrinological evidence is creating a conundrum for researchers. This review summarizes what is known about sample characteristics reported in neuroimaging, endocrinological, and genetics research into romantic love and presents ideas for future studies. It is important to have a harmonized approach to sample reporting to maximize comparability between studies and the ability to determine findings’ generalizability.

First, we define romantic love. Second, we detail the commonly used measures of romantic love. Third, we describe the methods we employed in this review. Fourth, we describe the sample characteristics reported in neuroimaging, endocrinological, and genetics studies. Fifth, we discuss the finding by summarizing the sample reporting practices in studies investigating the biological mechanisms of romantic love, introduce the concept of comparability and make an assessment of the comparability of existing studies, introduce the concept of generalizability and make an assessment of generalizability of existing studies, and suggest ideas for future research. These ideas could be adopted across the full spectrum of disciplines scientifically investigating romantic love.

## 2. Definition of romantic love

“Romantic love is a motivational state typically associated with a desire for long-term mating with a particular individual. It occurs across the lifespan and is associated with distinctive cognitive, emotional, behavioral, social, genetic, neural, and endocrine activity in both sexes. Throughout much of the life course, it serves mate choice, courtship, sex, and pair-bonding functions. It is a suite of adaptations and by-products that arose sometime during the recent evolutionary history of humans” (Bode and Kushnick, 2021, p. 21). Romantic love is sometimes referred to as “passionate love” and differs from companionate love, which is felt less intensely and often follows a period of romantic love (see Hatfield and Walster, 1985).

## 3. Commonly used measures of romantic love

### 3.1. Passionate love scale (PLS)

The PLS (Hatfield and Sprecher, 1986) is a 30-item or 15-item measure of the cognitive, emotional, and behavioral characteristics of romantic love in people who are in a romantic relationship. Cognitive items assess intrusive thinking or preoccupation with the partner, idealization of the other in the relationship, and desire to know the other and to be known by the other. Emotional items assess attraction to the other, especially sexual attraction, negative feelings when things go awry, longing for reciprocity, desire for complete union, and physiological arousal. Behavioral items assess actions toward determining the other’s feelings, studying the other person, service to the other, and maintaining physical closeness. Items were selected from among 165 items using item analysis in two studies. Examples of questions include “Since I’ve been involved with ____________, my emotions have been on a roller coaster,” “Sometimes I feel I cannot control my thought; they are obsessively on ____________,” and “I will love ____________ forever.”

Responses are measured on a 9-point Likert scale ranging from 1 (not at all true) to 9 (definitely true). Scores can range from 30 to 270 for the 30-item measure or 15–135 for the 15-item measure. The PLS was constructed and validated in two studies of 136 and 164 American university students involved in dating or more serious relationships. Alpha was 0.94 for the 30-item measure and 0.91 for the 15-item measure. A factor analysis indicated that the PLS assesses a single factor which explained 70% of the variance in that data. The PLS has been used widely cross-culturally (Feybesse and Hatfield, 2019) and has shown consistently high reliability in studies of romantic love (Graham and Christiansen, 2009).

### 3.2. Triangular love scale (TLS)

The TLS (Sternberg, 1988, 1997) is a 45-item measure of three dimensions of love (i.e., intimacy, passion, and commitment) identified in the triangular theory of love (Sternberg, 1986) in people who are in a social relationship. In the context of romantic love, it should be administered to individuals who are in a romantic relationship. Each subscale includes 15 items and is scored separately. The intimacy subscale assesses feelings of closeness, connectedness, and bondedness. The passion subscale assesses drives that lead to romance, physical attraction, and sexual consummation. The commitment subscale assesses the decision that an individual love’s their partner and their commitment to maintaining the relationship. Examples of questions include “I am able to count on ____________ in times of need,” “I cannot imagine life without ____________,” and “I feel a sense of responsibility toward ____________.”

Responses are measured on a 9-point Likert scale ranging from 1 (not at all) to 9 (extremely). Scores on each subscale can range from 15 to 135. Thresholds for each subscale indicate whether an individual is experiencing that component of love from significantly below average to significantly above average. Romantic love is associated with high scores on passion and intimacy subscales, but commitment can also be high (in which case it is called consummate love). A 36-item TLS was validated in two studies of 84 and 101 American adults recruited via newspaper advertisements (Sternberg, 1997). Alphas were 0.91 for intimacy, 0.94 for passion, 0.94 for commitment, and 0.97 overall. Factor analysis indicated that a three-factor solution explained 60% of the variance in the data. The TLS has high reliability across studies with different sample characteristics (Graham and Christiansen, 2009) and has been demonstrated to be psychometrically sound cross-culturally (Sorokowski et al., 2021). A number of attempts have been made to develop a short-form of the TLS (e.g., Kowal et al., 2022).

### 3.3. Eros subscale of the love attitudes scale (LAS)

The LAS (Hendrick and Hendrick, 1986) is a measure of love attitudes based on the “colors of love” theory (Lee, 1973). It is a 42-item scale assessing six types of love styles: Mania (possessive, dependent love), Eros (romantic love), Pragma (logical, “shopping list” love), Storge (friendship love), Ludus (game-playing love), or Agape (all-giving, selfless love). It was not intended to measure romantic love but has increasingly been used as a measure of individuals’ experience of love (Hatfield et al., 2012), and is sometimes used as a measure of romantic love. The Eros subscale is a 7-item measure assessing emotions, behaviors, aspects of attraction, and relationship characteristics. Its focus is on strong physical preferences, early attraction, intensity, and commitment to an individual’s lover. It asks questions that can only be administered to individuals who are in a romantic relationship. Examples of questions include “My lover and I were attracted to each other immediately after we first met,” “Our lovemaking is very intense and satisfying,” and “My lover fits my ideal standards of physical beauty/handsomeness.”

Responses are measured on a 5-point Likert scale ranging from 1 (strongly agree) to 5 (strongly disagree). The LAS was originally validated in two studies of 807 American university students and 567 American University students. Alpha for the Eros subscale was 0.70. Factor analysis demonstrated suitable internal reliability, and reasonable independence from the other LAS subscales. A 4-item and 3-item short form of the Eros subscale has been developed (Hendrick et al., 1998) with alphas of 0.82 and 0.79, respectively.

### 3.4. Time thinking about a loved one

Time spent thinking about a loved one is a single item assessment of romantic love. Obsessive thinking about a loved one is characteristic of early-stage romantic love (see Hatfield and Sprecher, 1986; Fisher, 1998; Leckman and Mayes, 1999) but this is not the case in long-term romantic love (Acevedo and Aron, 2009; O’Leary et al., 2011). It can be assessed in terms of number of hours per day spent thinking about a loved one (e.g., Marazziti et al., 1999; Marazziti and Canale, 2004; Emanuele et al., 2006) or percentage of waking hours spent thinking about a loved one (e.g., Langeslag et al., 2012). We are not aware of any studies that have validated this item as a measure of romantic love. Nonetheless, time spent thinking about a loved one is a simple and short means of assessing romantic love, regardless of relationship status.

### 3.5. A validated measure suitable for individuals not in a relationship with their loved one (infatuation scale)

Unrequited love is a neglected phenomenon in biological mechanisms research into romantic love. It has been identified as an important area for future research (Bode and Kushnick, 2021). Unrequited love takes a number of forms: love for a celebrity, love for someone known but not actively pursued, love for someone known being pursued, love for a former partner, and unequal love relationship (Bringle et al., 2013). All but one of these (i.e., unequal love relationship) are individuals who are not in a romantic relationship with their loved one.

The Infatuation Scale (Langeslag et al., 2012) is a 10-item measure of romantic infatuation. Infatuation is one component of romantic love (see the attraction system in Fisher, 1998, 2000; Fisher et al., 2002). The Infatuation Scale is suitable for administration to individuals in romantic relationships and individuals not in romantic relationships. The Infatuation Scale assesses cognitive, emotional, behavioral, and physiological characteristics associated with infatuation. Examples of questions include “I get shaky knees when I am near ______,” “My thoughts about ______ make it difficult for me to concentrate on something else,” and “I am shy in the presence of ______.”

Responses are measured on a 7-point Likert scale ranging from 1 (strongly agree) to 7 (strongly disagree). Scores can range from 10 to 70. The Infatuation Scale was constructed and validated in conjunction with the Attachment Scale in three studies of 162 Dutch-speakers who were in love and/or were involved in a romantic relationship, 214 Dutch-speakers who were in love and/or were involved in a romantic relationship, and 183 English-speakers who were in love and/or were involved in a romantic relationship. Alphas for the Infatuation Scale in each study were 0.94, 0.89, and 0.83, respectively. One exploratory factor analysis and two confirmatory factor analyses indicated that the Infatuation Scale constituted one of two factors (the second being the Attachment Scale). Infatuation Scale scores tend to be higher in individuals who are not in a romantic relationship with their loved one compared with those who are in a romantic relationship with their loved one. The Infatuation Scale has not been widely adopted in the literature. The Infatuation Scale may not be suitable for all types of unrequited love (i.e., love for a celebrity) and may not be discriminating in people who love a past lover, but it is the only validated measure related to romantic love that we are aware of that is suitable for the majority of people who are not in a relationship with their loved one.

## 4. Methods

We undertook three separate searches for studies investigating the mechanisms of romantic love (i.e., neuroimaging studies, endocrinological studies, and genetics studies). To identify relevant neuroimaging studies, we undertook three separate topic searches on Web of Science and three separate title/abstract searches of PubMed to identify relevant neuroimaging studies. We searched for (i) love AND EEG, (ii) love AND ERP, (iii) love AND fMRI, and (iv) love AND “positron emission tomography.” We also included other studies that we were aware of and contacted relevant authors to see if they could identify any relevant studies we had missed. To identify relevant endocrinological studies, we collated all the endocrine studies of romantic love that we were aware of, including those identified from reading the text of relevant studies. We also contacted relevant authors to see if they could identify any relevant studies we had missed. To identify relevant genetics studies, we undertook a topic search on Web of Science and a title/abstract search on PubMed to identify relevant genetics. We searched for love AND genetic. We also contacted relevant authors to see if they could identify any relevant studies we had missed. Inclusion criteria were studies published in peer-reviewed articles up to and including 2021 with a group or entire sample experiencing romantic love.

## 5. Sample characteristics reported in studies

### 5.1. Neuroimaging studies

We identified 32 neuroimaging studies (i.e., 10 EEG/ERP, 21 fMRI, 1 PET) involving 688 particpants experiencing romantic love reported in 29 peer-reviewed journal articles that met our inclusion criteria. Table 1 presents the neuroimaging studies included in this review. Supplementary Table 1 presents a summary of the romantic love sample characteristics reported in neuroimaging studies with a group or entire sample experiencing romantic love.

**Table 1:** Neuroimaging studies included in this review (reference, number of subjects in the romantic love groups, and type of imaging undertaken).

References	*n*	Type of imaging
Bartels and Zeki (2000)	17	fMRI
Aron et al. (2005)	17	fMRI
Langeslag et al. (2007)	18	EEG
Ortigue et al. (2007)	36	fMRI
Langeslag et al. (2008)	20	EEG
Kim et al. (2009)	10	fMRI
Fisher et al. (2010)	15	fMRI
Younger et al. (2010)	15	fMRI
Zeki and Romaya (2010)	24	fMRI
Xu et al. (2011)	18	fMRI
Stoessel et al. (2011)	12	fMRI
Cacioppo et al. (2012)	20	EEG
Cannas Aghedu et al. (2021)	22	EEG
Acevedo et al. (2012)	17	fMRI
Xu et al. (2012a)	18	fMRI
Xu et al. (2012b)	12	fMRI
Scheele et al. (2013)	S1: 20	fMRI
Scheele et al. (2013)	S2: 20	fMRI
Yin et al. (2013)	36	fMRI
Langeslag et al. (2014)	15	fMRI
Langeslag et al. (2015)	S1: 20	EEG
Langeslag et al. (2015)	S2: 18	EEG
Song et al. (2015)	34	fMRI
Takahashi et al. (2015)	10	PET
Langeslag and van Strien (2016)	S1: 32	EEG
Wang et al. (2016)	22	fMRI
Yin et al. (2018)	32^4^	fMRI
Langeslag and Van Strien (2019)	24	EEG
Acevedo et al. (2020)	T1: 19, T2: 13	fMRI
Langeslag and van Strien (2020)	S1: 24	EEG
Langeslag and van Strien (2020)	S2: 24	EEG
Wang et al. (2020)	34	fMRI

EEG, Electroencephalogram; fMRI, Functional magnetic resonance imaging; PET, Positron emission topography; S1, Study 1; S2, Study 2; T1, Time 1; T2, Time 2.

All studies reported the sample size of participants experiencing romantic love. All but one study reported the gender of participants. All but two studies reported the mean age of participants and many reported the age range. Twenty-seven studies used the Passionate Love Scale (PLS; Hatfield and Sprecher, 1986) as a measure of romantic love. Some used the 30-item version and some used the 15-item version (it is not possible to determine precisely how many studies used each version because most studies do not indicate which version was used and only report mean item scores instead of total scores). Four studies used the Infatuation Scale and the Attachment Scale (Langeslag et al., 2012). Twenty studies reported mean relationship duration and most of these reported the relationship duration range. Sixteen studies reported mean length of time in love and some reported the range. All studies provided additional descriptors of the participants (e.g., sociodemographic or health characteristics). Only 16 studies explicitly provided the country in which the study took place, although this information can be indirectly inferred from, for instance, authors’ affiliations or information on which ethical committee approved the study’s protocols.

### 5.2. Endocrinological studies

We identified nine endocrinological studies involving 364 participants experiencing romantic love that met our inclusion criteria. Table 2 presents the endocrinological studies included in this review. Supplementary Table 2 presents a summary of the romantic love sample characteristics reported in endocrinological studies with a group or entire sample experiencing romantic love.

**Table 2:** Endocrinological studies included in this review (reference, number of subjects in the romantic love groups, and factors measured).

References	*n*	Factors
Marazziti et al. (1999)	20	Serotonin transporter
Marazziti and Canale (2004)	24	FSH, LH, estradiol, progesterone, DHEA, cortisol, testosterone, androstenedione
Emanuele et al. (2006)	58	NGF
Dundon and Rellini (2012)	29	Norepinephrine, dopamine
Langeslag et al. (2012)	20	Serotonin
Weisman et al. (2015)	120	Cortisol
Marazziti et al. (2017)	30	Dopamine transporter
Sorokowski et al. (2019)	47	Estradiol, LH, FSH, prolactin, testosterone, cortisol
Renner et al. (2021)	16	Cortisol, DHEA, progesterone

FSH, follicle stimulating hormone; LX, luteinizing hormone; DHEA, dehydroepiandrosterone.

All studies reported the sample size of participants experiencing romantic love. All studies reported the gender of participants. All but one study reported the age of participants (mostly the mean and standard deviation). A number of ways of measuring romantic love were used. Four studies used the number of hours spent thinking about the loved one, three studies used the PLS, and two studies used the Triangular Love Scale (TLS; Sternberg, 1988) or a subscale of the TLS (although one study did not report the results). Five studies reported the mean relationship duration and six studies reported the permitted relationship duration for inclusion in the study. All studies reported sample descriptors, although, once again, these are often provided only as inclusion or exclusion criteria. One study reported the country in which the study took place and one study reported the city. However, some inferences can be made from the languages of certain measures in some studies, the source of ethics approval, or the affiliations of authors.

### 5.3. Genetics studies

We identified two genetics studies involving, in total, 36 participants experiencing romantic love that met our inclusion criteria. Table 3 presents the genetics studies included in this review. Supplementary Table 3 presents a summary of the romantic love sample characteristics reported in genetics studies with a group or entire sample experiencing romantic love.

**Table 3:** Genetic studies included in this review (reference, number of subjects in the romantic love groups, genetic characteristic assessed).

References	*n*	Assessed characteristic
Murray et al. (2019)	17	Immune cell gene regulation (115 genes)
Acevedo et al. (2020)	T1: 19, T2: 13	AVPR1a rs3, OXTR rs53576, COMT rs4680, and DRD4-7R alleles

T1, Time 1; T2, Time 2.

Both studies reported the sample size of participants experiencing romantic love and the gender of participants. One study reported the age of the participants (experiencing romantic love) while the other only reported the age of the entire sample (including non-romantic love participants). One study measured romantic love using the Eros subscale of the Love Attitudes Scale (LAS; Hendrick and Hendrick, 1986) while the other study relied on self-reported experience of having fallen in love. We are aware of one other genetics study that did not meet our inclusion criteria which used the Eros subscale of the LAS (Emanuele et al., 2007). One study reported relationship duration while the other indirectly indicated relationship duration (a combination of range at baseline plus median at follow-up). Both studies provided descriptors of the sample. One study indicated the nationality of participants.

## 6. Discussion

In the sections above, we defined romantic love, summarized the sample characteristics reported in neuroimaging, endocrinological, and genetics studies. Now, we will summarize the sample reporting practices of studies investigating the biological mechanisms of romantic love, detail the resulting implications for comparability and generalizability, and present ideas for sample reporting in future studies.

### 6.1. Summary of sample reporting practices

All but one relevant study reported the sample size of people experiencing romantic love and the gender of participants. All but one relevant study reported the sample age of participants. This is frequently done by reporting the range, mean, and standard deviation. Almost all studies reported the gender of participants. This was probably done because gender can sometimes serve as a proxy for sex. Neuroimaging studies, overwhelmingly, used the PLS, one relevant genetics study used the Eros subscale of the LAS, and endocrinological studies used a variety of measures. Studies used the long-form and short-form of PLS. Studies frequently reported the mean and standard deviation of total scores and mean items scores on measures of romantic love. Most studies reported relationship duration and/or time in love (by reporting the range, mean, and standard deviation, although some simply report the range or relationship duration permitted for inclusion in the study). Almost all studies reported additional descriptors of participants, although, often, this was done because they detailed inclusion and exclusion criteria. Few studies reported extensive sociodemographic or health-related descriptors. Some studies reported the country in which the study took place.

### 6.2. Comparability

The reporting practices of studies investigating the biological mechanisms in romantic love have implications for the comparability of studies. This is true for comparing studies both between and among different types of mechanism research (i.e., neuroimaging, endocrinological, genetics). Comparability is important because it situates each individual study within the context of similar research and ensures that studies are investigating the same phenomenon in the same types of situations. Having comparable samples permits an assessment of the total weight of evidence for findings. Comparing the sample sizes, sexes, and ages of participants across included studies is simple enough because these data are reported for almost all studies. Comparing the romantic love characteristics, however, is more challenging. Comparability of samples’ romantic love characteristics is of the utmost importance, because it permits an assessment of whether all studies are investigating the same phenomenon.

There are five ways of assessing romantic love used in the studies that we consider (i.e., PLS, TLS, LAS, time spent thinking about loved one, self-report), and comparing studies’ results is a fruitless endeavor. The love scales (PLS, passion and intimacy subscales of the TLS, and Eros subscale of the LAS) were found to be moderately correlated with each other (with *r* ranges from 0.49 to 0.79; Graham, 2011). The TLS does not provide a single measure of romantic love, but rather provides a measure of three different components of love, two of which are said to be involved in romantic love. Further, the Eros subscale of the LAS assesses a type of romantic love associated with a rapid attraction onset and emotional involvement–things that are inconsistent with the way the majority of romantic relationships develop (see Stinson et al., 2021). These discrepancies are particularly problematic when comparing studies with contrasting findings or when comparing studies or different mechanisms.

Most studies report relationship duration or time in love. However, these two indicators are not equivalent. The onset of romantic love and the formation of a romantic relationship are likely to occur within temporal proximity, but are distinct phenomena. Romantic love may precede or follow the formation of a relationship. While both factors are relevant and potentially important in understanding romantic love, uniformity would improve the comparability of studies.

Finally, the descriptors of study participants in relevant studies are not sufficient to ensure comparability. Most studies’ descriptors are consequences of reporting inclusion and exclusion criteria, rather than intentionally attempting to describe participants. This is particularly problematic in endocrinological research, where findings are inconsistent. Specifically, studies do not provide consistent findings concerning cortisol (Marazziti and Canale, 2004; Weisman et al., 2015; Sorokowski et al., 2019; Renner et al., 2021), testosterone (Marazziti and Canale, 2004; Sorokowski et al., 2019), and serotonin (Marazziti et al., 1999; Langeslag et al., 2012). Other disciplines (e.g., epidemiology) regularly and consistently report the socio-demographic characteristics of samples, and this adds substantially to the comparability of samples and findings across studies. Romantic love research may learn from other such disciplines. Increasing the comparability of sample characteristics would help to make better sense of inconsistent findings in future studies.

#### 6.2.1. Comparability of existing studies

Having outlined the importance of ensuring comparability of studies, it is necessary to also make a determination about the comparability of existing studies investigating the mechanisms of romantic love. Almost all studies report some basic information, such as age of participants, sex of participants, and a measure of romantic love. A number of studies can be compared because they report similar data characteristics (such as time in love or relationship duration). In our assessment, however, comparability between all studies is only possible in terms of age and sex (although neuroimaging studies can largely be compared in terms of scores on the Passionate Love Scale). Comparing samples of all studies on more than this is not possible.

### 6.3. Generalizability

The sample reporting practices of studies investigating the biological mechanisms of romantic love have implications for the generalizability of studies. We deem generalizability as crucial because it can determine the degree to which findings apply to a particular group or groups or are indicative of a universal phenomenon. The issues at hand are whether findings are likely to apply, generally or specifically, to other study settings or samples. Can the findings be applied to all humans experiencing romantic love? To answer this, we must consider sampling and representativeness, the potential for bias, and confounding factors, and how the reporting of sample characteristics can help us manage these influences (Kukull and Ganguli, 2012).

The romantic love groups used in biological mechanisms studies tend to be self-selected convenience samples. That is, using advertising or other means, individuals become aware of an opportunity to take part in the research. University students are commonly used in these studies. There are obvious benefits to this approach, namely, making the recruitment process efficient and ensuring participants are committed to the study. However, there are a number of problems, including self-selection bias and non-representativeness of the sample (Sharma, 2017). This unrepresentativeness could potentially give rise to a selection bias. The specific types of people willing to participate in the research possess characteristics that make the results less applicable to the general population (Henderson and Page, 2007). This selection bias could mean that certain factors confound the results. In the case of romantic love, personality or motivation may be different in people willing to engage in romantic love or relationship research.

The features of samples in studies investigating the biological mechanisms of romantic love should also be considered in a broader context. Researchers have highlighted the general bias in scientific research to use Western, educated, industrialized, rich and democratic (WEIRD) samples (Henrich et al., 2010; Apicella et al., 2019). This is certainly the case in relation to the studies we consider. Although the nation in which the studies were conducted is not reported in most studies, consideration of the universities to which authors are affiliated and the source of ethics approvals indicates that the overwhelming majority of relevant studies take place in WEIRD countries, particularly, the USA. Western, educated, industrialized, rich, and developed populations differ from other populations on a range of psychological characteristics and traits. At present, we cannot say if this is the case in relation to some biological features associated with romantic love. Romantic love is very similar cross-culturally, but some differences do exist (see Sprecher et al., 1994; Feybesse and Hatfield, 2019; Karandashev, 2019). Progressing biological mechanisms research to a more nuanced level of analysis requires consideration that differences might exist between populations. To do this, it is necessary to ensure sufficient description of samples to permit assessment of how WEIRD each sample is.

Robust and detailed reporting is not going to fix limited generalizability. However, it can help to mitigate this problem by providing detailed descriptions of participant characteristics against which the broader population can be compared. Sex and age are important, but greater emphasis needs to be placed on the consistent reporting of romantic love characteristics as well as sociodemographic, psychological, and health characteristics. This will enable readers to assess the likelihood that findings can be generalized to a broader population.

#### 6.3.1. The generalizability of existing studies

In light of the importance we have placed on an ability to assess the generalizability of findings, it seems appropriate to comment on the generalizability of existing studies. Limited sample descriptions in most studies substantially limit our ability to assess the generalizability of existing studies. Lack of information about geographical location in some studies and socioeconomic status means that we cannot assert that findings are representative of the general population in any particular country or globally. The high proportion of studies using university students and participants of a relatively young age also suggests that the findings may be particular to a younger population.

### 6.4. Ideas for future research

**Figure 1:**
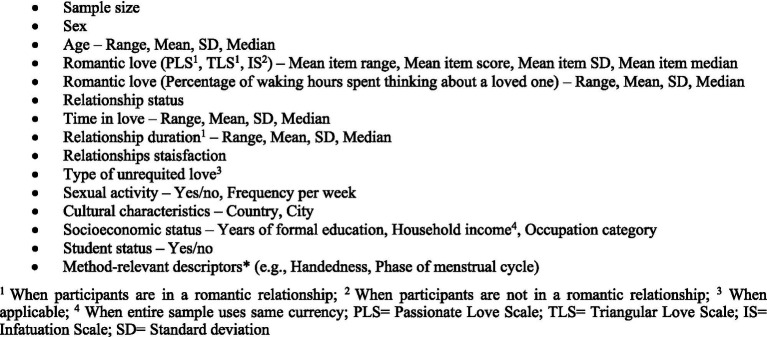
Ideas for sample characteristics to be reported in studies investigating the biological mechanisms of romantic love.

In light of the sample reporting practices detailed above, we present ideas about what sample characteristics should be used in future studies to improve comparability and the ability to assess generalizability. Figure 1 presents ideas for characteristics to be reported in future studies investigating the biological mechanisms of romantic love.

#### 6.4.1. Sex

Existing studies rarely report sex; they use gender as a proxy for sex. The proliferation of non-traditional gender identities in recent years means that researchers will face an increasing problem using gender as a proxy for sex. As a result, we believe that future studies could, at a minimum, report the biological sex of participants. Sex is either male or female. An “intersex” category may be required in some circumstances. Further guidance related to the reporting of sex can be found in the Sex and Gender Equity Research (SAGER) guidelines (see Heidari et al., 2016).

#### 6.4.2. Age

Romantic love occurs across the lifespan (Hatfield et al., 1988; Wang and Nguyen, 1995), but different ages are associated with different frequencies and characteristics. To maximize comparability across studies, age range, mean, SD, and median could be reported.

#### 6.4.3. Romantic love characteristics

Existing studies used multiple measures of romantic love. We believe that all studies would benefit from reporting results for the PLS and TLS (i.e., where individuals are in a romantic relationship). The PLS is the most commonly used measure in biological mechanisms research of romantic love. However, it focuses mainly on the passionate component of love (Hatfield and Sprecher, 1986), often associated with limerence (Tennov, 1979). Individuals can have similar scores but have radically different experiences (Cannas Aghedu et al., 2019). The TLS, on the other hand, captures not only the passionate aspect of love, but also intimacy and commitment (Sternberg, 1986), which were distinguished as related to the PLS but not equivalent to it (Hatfield and Sprecher, 1986). Thus, the TLS provides much- needed additional information that may have implications for study results. Full-length or short-forms would be appropriate, but which version is used should be reported. There will also be a need to measure romantic love in individuals not in a relationship with their loved. Where such participants exist, the Infatuation Scale should be administered. If the specific measure is detailed, and used the normal scoring approach, it would only be necessary to report mean item score range, mean item score, mean item score standard deviation, and median. The percentage of waking hours spent thinking about a loved one should also be included in all studies assessing the biological mechanisms of romantic love (see Langeslag et al., 2012).

#### 6.4.4. Relationship status

Studies would benefit from reporting the relationship status of participants in studies of romantic love. Possible status categories could include the following: (i) not in a romantic relationship, (ii) dating and not cohabiting, (iii) in a committed romantic relationship but not cohabiting, (iv) in a committed romantic relationship and cohabiting, or (v) married or *de facto* (see Langeslag et al., 2012).

#### 6.4.5. Relationship duration

Relationship duration is more commonly reported in relevant studies than time in love. Relationship duration is potentially informative because it serves as a proxy for the stage of romantic love (Garcia, 1997). As a result, studies would benefit from reporting the relationship duration of participants where possible.

#### 6.4.6. Time in love

Romantic love not in a relationship is more common than romantic love in a relationship in young adults and adolescents (see Bringle et al., 2013; Kuula et al., 2020). Unrequited love has been identified as an important area of future research (Bode and Kushnick, 2021). As a result, promoting comparability among future studies may require the reporting of time in love. We believe that studies would benefit from reporting time in love.

#### 6.4.7. Relationship satisfaction

Relationship satisfaction may be a cause or consequence of romantic love and has implication for how romantic love is experienced, especially in cases of uncertain relationship status or unrequited love. Being “happily” in love has been used as discriminate variable in some neuroimaging studies of romantic love (e.g., Stoessel et al., 2011). Relationship satisfaction can assess this feature. Relationship satisfaction can be measured with a single item asking “In general, how satisfied are you with your relationship?” (Fülöp et al., 2020). There are, however, other means (see Nichols et al., 1983; Schumm et al., 1986; Hendrick et al., 1998; Fletcher et al., 2000).

#### 6.4.8. Type of unrequited love

Few studies (e.g., Fisher et al., 2010; Stoessel et al., 2011; Song et al., 2015) have investigated the mechanisms associated with unrequited love. However, this is a necessary part of future biological mechanisms research into romantic love (Bode and Kushnick, 2021). Studies where unrequited lovers are participants would benefit from reporting the type of unrequited love. These may include: love for a celebrity, love for someone known but not actively pursued, love for someone known being pursued, love for former partner, unequal love relationship (Bringle et al., 2013). Determining unrequited love may involve asking participants if their loved one loves them in return.

#### 6.4.9. Sexual activity

Sexual activity (or the absence of sexual activity) is reported in a small number of the studies we consider (e.g., Marazziti et al., 1999; Marazziti and Canale, 2004; Acevedo et al., 2020). This is under-reported in biological mechanisms research given the close relationship between romantic love and sexual behavior. Sex is one of the functions of romantic love (Meston and Buss, 2007, 2009; Bode and Kushnick, 2021) and sexual behavior could potentially impact the mechanisms that are being measured in relevant studies (as alluded to in Meltzer et al., 2017). For example, oxytocin, a hormone that is released during sexual activity (Murphy et al., 1987; Carter, 1992), plays a role in social bonding (Olff et al., 2013) and romantic attachment, especially at the initial stages of romantic attachment (Schneiderman et al., 2012). Neural structures rich in oxytocin receptors are active in people experiencing romantic love (e.g., Bartels and Zeki, 2004). As a result, studies would benefit from reporting if an individual is engaging in sexual activity with their partner or loved one and the frequency of this sexual activity (per week; e.g., Acevedo et al., 2020).

#### 6.4.10. Cultural characteristics

Some studies report the cultural or ethnic characteristics of participants. Culture plays a role in the experience and expression of romantic love (Karandashev, 2019), although the psychological characteristics, measured by commonly used scales, are remarkably similar (Feybesse and Hatfield, 2019). Studies would benefit from reporting the country in which studies occur. Reporting the city in which studies take place may also be useful as differences in culture and language can exist within countries. The relevant factor here is where the sample comes from.

#### 6.4.11. Socioeconomic status

“Socioeconomic factors and social class are fundamental determinants of human functioning across the life span, including development, well-being, and physical and mental health” (American Psychological Association Task Force of Socioeconomic Status, 2007, p. 1). It is a construct often derived from a combination of education, income, and occupation. Assessing socioeconomic status is particularly important to ensure that an assessment of generalizability of findings can be undertaken. To maximize comparability between studies, a single item assessing years of formal education could be used. Household income could be collected when all participants are from within one country or economic zone (i.e., use the same currency). Guidance for occupation categories may be taken from the International Standard Classification of Occupations (i.e., managers, professionals, technicians and associated professionals, clerical support workers, service and sales workers, skilled agricultural, forestry, and fishery workers, craft and related trades workers, plant and machine operators and assemblers, elementary occupations, and armed forces occupations; Tripartite Meeting of Experts on Labour Statistics on Updating the International Standard Classification of Occupations, 2007).

#### 6.4.12. Student status

One of the main criticisms of some research is that it relies heavily on university students (Henrich et al., 2010). University students are an easily accessible and cooperative population with which to conduct biological mechanisms research into romantic love. However, it is important to acknowledge that using this population may limit the generalizability of findings. Studies would benefit from making clear what proportion of their samples are students.

#### 6.4.13. Method-relevant descriptors

There are circumstances where specific characteristics are relevant to the study design. For example, right-handedness is relevant to neuroimaging studies and phase (or day) of menstrual cycle is relevant to some endocrine studies. Studies would benefit from reporting these types of characteristics, when relevant.

#### 6.4.14. Reporting additional sample characteristics

Our ideas outline what sample characteristics we believe all biological mechanisms studies of romantic love should report. However, these represent just the minimum characteristics that should be reported. We encourage researchers to report a greater number of characteristics. Characteristics that would be particularly useful for promoting comparability or an assessment of generalizability include gender identity and sexual orientation, number of times ever in love, additional measures of relationship duration (i.e., how long known the loved one), sexual activity (i.e., time since last sex), more detailed cultural characteristics (i.e., country in which participant spent most of their childhood) or ethnicity, day of menstrual cycle for females, as well as a number of variables related to relationship dissolution, when relevant (i.e., time since breakup, initiator status), number and age of children, when relevant, and health characteristics (i.e., physical and mental health status). Researchers may also choose to administer the Infatuation Scale to all individuals in a relationship, as well as to individuals not in a romantic relationship, permitting a comparison of findings across relationship status. We refer readers to an endocrine study by Renner et al. (2021) as a good example of how extensive sample characteristics reporting can enrich a study and provide a deeper understanding of the findings.

### 6.5. Implications for other types of studies

The focus of this review is the sample characteristics reported in studies investigating the biological mechanisms of romantic love. Our ideas relate specifically to these types of studies. However, we suggest that other types of research into romantic love, such as psychological research, would also benefit from adopting our ideas. This would help to promote comparability among psychological studies as well as their generalizability, and also promote comparability between psychological and biological mechanisms studies.

## 7. Conclusion

This article lays out an argument and ideas for consistent sample reporting practices in biological mechanisms research of romantic love. We find that comparing studies is difficult because of inconsistent use of measures of romantic love as well as limited reporting of additional relevant characteristics. Assessments of generalizability are also hampered because of a lack of descriptive information that allows a comparison with the general population or specific populations. We provided ideas for sample reporting characteristics that will enhance comparability of studies and the ability to make an assessment of generalizability (i.e., sample size, sex age, romantic love, relationship status, time in love, relationship duration, relationship satisfaction, type of unrequited love, sexual activity, cultural characteristics, socioeconomic status, student status, and method-relevant descriptors).

First, we defined romantic love. Second, we detailed the commonly-used measures of romantic love. Third, we described the methods we employed in this review. Fourth, we described the sample characteristics reported in neuroimaging, endocrinological, and genetics studies. Fifth, we discussed the finding by summarizing the sample reporting practices in studies investigating the biological mechanisms of romantic love, introduce the concept of comparability and make an assessment of the comparability of existing studies, introduce the concept of generalizability and make an assessment of generalizability of existing studies, and suggest ideas for future research. These ideas can be applied, in part or in whole, to studies that use designs other than that of a controlled comparison. They may also be used in studies employing alternative or more complex designs. While our focus has been to describe and present ideas for sample reporting characteristics in biological mechanisms research into romantic love, these ideas may be employed effectively in other types of research.

## Author contributions

AB conceived and wrote the article. MK critically reviewed the manuscript several times, contacted relevant authors to identify studies for inclusion, and made a substantial intellectual contribution by helping to decide on the ideas made in the article. All authors contributed to the article and approved the submitted version.

## Conflict of interest

The authors declare that the research was conducted in the absence of any commercial or financial relationships that could be construed as a potential conflict of interest.

## Publisher’s note

All claims expressed in this article are solely those of the authors and do not necessarily represent those of their affiliated organizations, or those of the publisher, the editors and the reviewers. Any product that may be evaluated in this article, or claim that may be made by its manufacturer, is not guaranteed or endorsed by the publisher.

## References

[ref1] Acevedo B. P. Aron A. (2009). Does a long-term relationship kill romantic love? Rev. Gen. Psychol. 13, 59–65. doi: 10.1037/a0014226

[ref2] Acevedo B. P. Aron A. Fisher H. E. Brown L. L. (2012). Neural correlates of long-term intense romantic love. Soc. Cogn. Affect. Neurosci. 7, 145–159. doi: 10.1093/scan/nsq092, PMID: 21208991 PMC3277362

[ref3] Acevedo B. P. Poulin M. J. Collins N. L. Brown L. L. (2020). After the honeymoon: neural and genetic correlates of romantic love in newlywed marriages. Front. Psychol. 11:634. doi: 10.3389/fpsyg.2020.00634, PMID: 32457675 PMC7223160

[ref4] American Psychological Association Task Force of Socioeconomic Status. (2007). Report of the APA task force on socioeconomic status. Washington, DC: American Psychological Association.

[ref5] Apicella C. Norenzayan A. Henrich J. (2019). Beyond WEIRD: a review of the last decade and a look ahead to the global laboratory of the future. Evol. Hum. Behav. 41, 319–329. doi: 10.1016/j.evolhumbehav.2020.07.015

[ref6] Aron A. Fisher H. Mashek D. J. Strong G. Li H. F. Brown L. L. (2005). Reward, motivation, and emotion systems associated with early-stage intense romantic love. J. Neurophysiol. 94, 327–337. doi: 10.1152/jn.00838.2004, PMID: 15928068

[ref7] Bartels A. Zeki S. (2000). The neural basis of romantic love. Neuroreport 11, 3829–3834. doi: 10.1097/00001756-200011270-00046, PMID: 11117499

[ref8] Bartels A. Zeki S. (2004). The neural correlates of maternal and romantic love. Neuroimage 21, 1155–1166. doi: 10.1016/j.neuroimage.2003.11.003, PMID: 15006682

[ref9] Bode A. Kushnick G. (2021). Proximate and ultimate perspectives on romantic love. Front. Psychol. 12:573123. doi: 10.3389/fpsyg.2021.573123, PMID: 33912094 PMC8074860

[ref10] Bringle R. G. Winnick T. Rydell R. J. (2013). The prevalence and nature of unrequited love. SAGE Open 3:215824401349216. doi: 10.1177/2158244013492160

[ref11] Cacioppo S. Grafton S. T. Bianchi-Demicheli F. (2012). The speed of passionate love, as a subliminal prime: a high-density electrical neuroimaging study. Neuroquantology 10, 715–724. doi: 10.14704/nq.2012.10.4.509

[ref12] Cannas Aghedu F. Sarlo M. Zappasodi F. Acevedo B. P. Bisiacchi P. S. (2021). Romantic love affects emotional processing of love-unrelated stimuli: an EEG/ERP study using a love induction task. Brain Cogn. 151:105733. doi: 10.1016/j.bandc.2021.105733, PMID: 33915402

[ref13] Cannas Aghedu F. Veneziani C. Bisiacchi P. (2019). The multidimensional evaluation of love (MEVOL) scale: development and preliminary validation. Test. Psychom. Methodol. Appl. Psychol. 26, 249–269. doi: 10.4473/TPM26.2.6

[ref14] Carter C. S. (1992). Oxytocin and sexual behavior. Neurosci. Biobehav. Rev. 16, 131–144. doi: 10.1016/S0149-7634(05)80176-9 1630727

[ref15] Dundon C. M. Rellini A. H. (2012). Emotional states of love moderate the association between catecholamines and female sexual responses in the laboratory. J. Sex. Med. 9, 2617–2630. doi: 10.1111/j.1743-6109.2012.02799.x, PMID: 22621174

[ref16] Emanuele E. Brondino N. Pesent S. Re S. Geroldi D. (2007). Genetic loading on human loving styles. Neuroendocrinol. Lett. 28, 815–821. PMID: 18063936

[ref17] Emanuele E. Politi P. Bianchi M. Minoretti P. Bertona M. Geroldi D. (2006). Raised plasma nerve growth factor levels associated with early-stage romantic love. Psychoneuroendocrinology 31, 288–294. doi: 10.1016/j.psyneuen.2005.09.002, PMID: 16289361

[ref18] Feybesse C. Hatfield E. (2019). “Passionate love” in The new psychology of love. eds. Sternberg R. J. Sternberg K. 2nd ed (Cambridge: Cambridge University Press), 183–207.

[ref19] Fisher H. E. (1998). Lust, attraction, and attachment in mammalian reproduction. Hum. Nat. 9, 23–52. doi: 10.1007/s12110-998-1010-5, PMID: 26197356

[ref20] Fisher H. (2000). Lust, attraction, attachment: biology and evolution of the three primary emotion systems for mating, reproduction, and parenting. J. Sex Educ. Ther. 25, 96–104. doi: 10.1080/01614576.2000.11074334

[ref21] Fisher H. E. Aron A. Mashek D. Li H. Brown L. L. (2002). Defining the brain systems of lust, romantic attraction, and attachment. Arch. Sex. Behav. 31, 413–419. doi: 10.1023/a:1019888024255, PMID: 12238608

[ref22] Fisher H. E. Brown L. L. Aron A. Strong G. Mashek D. (2010). Reward, addiction, and emotion regulation systems associated with rejection in love. J. Neurophysiol. 104, 51–60. doi: 10.1152/jn.00784.2009, PMID: 20445032

[ref23] Fletcher G. J. O. Simpson J. A. Thomas G. (2000). The measurement of perceived relationship quality components: a confirmatory factor analytic approach. Personal. Soc. Psychol. Bull. 26, 340–354. doi: 10.1177/0146167200265007

[ref24] Fülöp F. Bőthe B. Gál É. Cachia J. Y. A. Demetrovics Z. Orosz G. (2020). A two-study validation of a single-item measure of relationship satisfaction: RAS-1. Curr. Psychol. 41, 2109–2121. doi: 10.1007/s12144-020-00727-y

[ref25] Garcia C. Y. (1997). Temporal course of basic components of love along the couple relationship. Psicothema 9, 1–15.

[ref26] Graham J. M. (2011). Measuring love in romantic relationships: a meta-analysis. J. Soc. Pers. Relat. 28, 748–771. doi: 10.1177/0265407510389126

[ref27] Graham J. M. Christiansen K. (2009). The reliability of romantic love: a reliability generalization meta-analysis. Pers. Relat. 16, 49–66. doi: 10.1111/j.1475-6811.2009.01209.x

[ref28] Hatfield E. Schmitz E. Cornelius J. Rapson R. L. (1988). Passionate love: how early does it begin? J. Psychol. Hum. Sex. 1, 35–51. doi: 10.1300/J056v01n01_04

[ref01] Hatfield E. Bensman L. Rapson R. L. (2012). A brief history of social scientists’ attempts to measure passionate love. J. Soc. Pers. Relatsh. 29, 143–164. doi: 10.1177/0265407511431055

[ref29] Hatfield E. Sprecher S. (1986). Measuring passionate love in intimate relationships. J. Adolesc. 9, 383–410. doi: 10.1016/S0140-1971(86)80043-4, PMID: 3805440

[ref30] Hatfield E. Walster G. W. (1985). A new look at love. Lanham, MD: University Press of America.

[ref31] Heidari S. Babor T. F. De Castro P. Tort S. Curno M. (2016). Sex and gender equity in research: rationale for the SAGER guidelines and recommended use. Res. Integr. Peer Rev. 1:2. doi: 10.1186/s41073-016-0007-6, PMID: 29451543 PMC5793986

[ref32] Henderson M. Page L. (2007). Appraising the evidence: what is selection bias? Evid. Based Ment. Health 10, 67–68. doi: 10.1136/ebmh.10.3.67 17652553

[ref33] Hendrick S. S. Dicke A. Hendrick C. (1998). The relationship assessment scale. J. Soc. Pers. Relat. 15, 137–142. doi: 10.1177/0265407598151009

[ref34] Hendrick C. Hendrick S. (1986). A theory and method of love. J. Pers. Soc. Psychol. 50, 392–402. doi: 10.1037/0022-3514.50.2.392

[ref35] Hendrick C. Hendrick S. S. Dicke A. (1998). The love attitudes scale: short form. J. Soc. Pers. Relat. 15, 147–159. doi: 10.1177/0265407598152001

[ref36] Henrich J. Heine S. J. Norenzayan A. (2010). The weirdest people in the world? Behav. Brain Sci. 33:61. doi: 10.1017/s0140525x0999152x, PMID: 20550733

[ref37] Karandashev V. (2019). Cross-cultural perspectives on the experience and expression of love Springer Nature.

[ref38] Kim W. Kim S. Jeong J. Lee K. U. Ahn K. J. Chung Y. A.. (2009). Temporal changes in functional magnetic resonance imaging activation of heterosexual couples for visual stimuli of loved partners. Psychiatry Investig. 6, 19–25. doi: 10.4306/pi.2009.6.1.19, PMID: 20046369 PMC2796039

[ref001] Kowal M. Sorokowski P. Dinić B. M. Pisanski K. Gjoneska B. Frederick D.. (2022). Validation of the Short Version (TLS-15) of the Triangular Love Scale (TLS-45) across 37 Languages. doi: 10.31234/osf.io/unea9 PMC10844340 37884798

[ref39] Kukull W. A. Ganguli M. (2012). Generalizability: the trees, the forest, and the low-hanging fruit. Neurology 78, 1886–1891. doi: 10.1212/WNL.0b013e318258f812, PMID: 22665145 PMC3369519

[ref40] Kuula L. Partonen T. Pesonen A. K. (2020). Emotions relating to romantic love-further disruptors of adolescent sleep. Sleep Health 6, 159–165. doi: 10.1016/j.sleh.2020.01.006, PMID: 32111523

[ref41] Langeslag S. J. E. Franken I. H. A. Van Strien J. W. (2008). Dissociating love-related attention from task-related attention: an event-related potential oddball study. Neurosci. Lett. 431, 236–240. doi: 10.1016/j.neulet.2007.11.044, PMID: 18162320

[ref42] Langeslag S. J. Jansma B. M. Franken I. H. Van Strien J. W. (2007). Event-related potential responses to love-related facial stimuli. Biol. Psychol. 76, 109–115. doi: 10.1016/j.biopsycho.2007.06.007, PMID: 17681417

[ref43] Langeslag S. Muris P. Franken I. (2012). Measuring romantic love: psychometric properties of the infatuation and attachment scales. J. Sex Res. 50, 739–747. doi: 10.1080/00224499.2012.714011, PMID: 23098269

[ref44] Langeslag S. Olivier J. Köhlen M. Nijs I. Van Strien J. (2015). Increased attention and memory for beloved-related information during infatuation: behavioral and electrophysiological data. Soc. Cogn. Affect. Neurosci. 10, 136–144. doi: 10.1093/scan/nsu034, PMID: 24526182 PMC4994849

[ref46] Langeslag S. J. E. van der Veen F. M. Fekkes D. (2012). Blood levels of serotonin are differentially affected by romantic love in men and women. J. Psychophysiol. 26, 92–98. doi: 10.1027/0269-8803/a000071

[ref47] Langeslag S. J. van der Veen F. M. Röder C. H. (2014). Attention modulates the dorsal striatum response to love stimuli. Hum. Brain Mapp. 35, 503–512. doi: 10.1002/hbm.22197, PMID: 23097247 PMC6869091

[ref48] Langeslag S. J. E. van Strien J. W. (2016). Regulation of romantic love feelings: preconceptions, strategies, and feasibility. PLoS One 11:e0161087. doi: 10.1371/journal.pone.0161087, PMID: 27529751 PMC4987042

[ref02] Langeslag S. J. E. van Strien J. W. (2019). Romantic love and attention: Early and late event-related potentials. Biol. Psychol. 146:107737. doi: 10.1016/j.biopsycho.2019.107737 31362051

[ref49] Langeslag S. J. E. van Strien J. W. (2020). Preferential processing of task-irrelevant beloved-related information and task performance: two event-related potential studies. Neuropsychologia 145:106497. doi: 10.1016/j.neuropsychologia.2017.09.015, PMID: 28927655

[ref50] Leckman J. F. Mayes L. C. (1999). Preoccupations and behaviors associated with romantic and parental love - perspectives on the origin of obsessive-compulsive disorder. Child Adolesc. Psychiatr. Clin. N. Am. 8:635. doi: 10.1016/S1056-4993(18)30172-X, PMID: 10442234

[ref51] Lee J. A. (1973). Colours of love: An exploration of the ways of loving. Toronto, ON: New Press.

[ref52] Marazziti D. Akiskal H. S. Rossi A. Cassano G. B. (1999). Alteration of the platelet serotonin transporter in romantic love. Psychol. Med. 29, 741–745. doi: 10.1017/s0033291798007946, PMID: 10405096

[ref53] Marazziti D. Baroni S. Giannaccini G. Piccinni A. Mucci F. Catena-Dell’Osso M.. (2017). Decreased lymphocyte dopamine transporter in romantic lovers. CNS Spectr. 22, 290–294. doi: 10.1017/s109285291600050x, PMID: 28031054

[ref54] Marazziti D. Canale D. (2004). Hormonal changes when falling in love. Psychoneuroendocrinology 29, 931–936. doi: 10.1016/j.psyneuen.2003.08.006, PMID: 15177709

[ref55] Meltzer A. L. Makhanova A. Hicks L. L. French J. E. McNulty J. K. Bradbury T. N. (2017). Quantifying the sexual afterglow: the lingering benefits of sex and their implications for pair-bonded relationships. Psychol. Sci. 28, 587–598. doi: 10.1177/0956797617691361, PMID: 28485699

[ref56] Meston C. M. Buss D. M. (2007). Why humans have sex. Arch. Sex. Behav. 36, 477–507. doi: 10.1007/s10508-007-9175-2 17610060

[ref57] Meston C. M. Buss D. M. (2009). Why women have sex. New York, NY: St Martin’s Griffin.

[ref58] Murphy M. R. Seckl J. R. Burton S. Checkley S. A. Lightman S. L. (1987). Changes in oxytocin and vasopressin secretion during sexual activity in men. J. Clin. Endocrinol. Metabol. 65, 738–741. doi: 10.1210/jcem-65-4-738, PMID: 3654918

[ref59] Murray D. R. Haselton M. G. Fales M. Cole S. W. (2019). Falling in love is associated with immune system gene regulation. Psychoneuroendocrinology 100, 120–126. doi: 10.1016/j.psyneuen.2018.09.043, PMID: 30299259 PMC6333523

[ref60] Nichols C. W. Schumm W. R. Schectman K. L. Grigsby C. C. (1983). Characteristics of responses to the Kansas marital satisfaction scale by a sample of 84 married mothers. Psychol. Rep. 53, 567–572. doi: 10.2466/pr0.1983.53.2.567

[ref61] O’Leary K. D. Acevedo B. P. Aron A. Huddy L. Mashek D. (2011). Is long-term love more than a rare phenomenon? If so, what are its correlates? Soc. Psychol. Personal. Sci. 3, 241–249. doi: 10.1177/1948550611417015

[ref62] Olff M. Frijling J. L. Kubzansky L. D. Bradley B. Ellenbogen M. A. Cardoso C.. (2013). The role of oxytocin in social bonding, stress regulation and mental health: an update on the moderating effects of context and interindividual differences. Psychoneuroendocrinology 38, 1883–1894. doi: 10.1016/j.psyneuen.2013.06.019, PMID: 23856187

[ref63] Ortigue S. Bianchi-Demicheli F. Hamilton A. Grafton S. T. (2007). The neural basis of love as a subliminal prime: an event-related functional magnetic resonance imaging study. J. Cogn. Neurosci. 19, 1218–1230. doi: 10.1162/jocn.2007.19.7.1218, PMID: 17583996

[ref64] Renner J. Stanulla M. Walther A. Schindler L. (2021). CortiLove: a pilot study on hair steroids in the context of being in love and separation. Compr. Psychoneuroendocrinol. 7:100061. doi: 10.1016/j.cpnec.2021.100061, PMID: 35757053 PMC9216709

[ref65] Scheele D. Wille A. Kendrick K. M. Stoffel-Wagner B. Becker B. Gunturkun O.. (2013). Oxytocin enhances brain reward system responses in men viewing the face of their female partner. Proc. Natl. Acad. Sci. U. S. A. 110, 20308–20313. doi: 10.1073/pnas.1314190110, PMID: 24277856 PMC3864312

[ref66] Schneiderman I. Zagoory-Sharon O. Leckman J. F. Feldman R. (2012). Oxytocin during the initial stages of romantic attachment: relations to couples’ interactive reciprocity. Psychoneuroendocrinology 37, 1277–1285. doi: 10.1016/j.psyneuen.2011.12.021, PMID: 22281209 PMC3936960

[ref67] Schumm W. R. Paff-Bergen L. A. Hatch R. C. Obiorah F. C. Copeland J. M. Meens L. D.. (1986). Concurrent and discriminant validity of the Kansas marital satisfaction scale. J. Marriage Fam. 48, 381–387. doi: 10.2307/352405

[ref68] Sharma G. (2017). Pros and cons of different sampling techniques. Int. J. Appl. Res. 3, 749–752.

[ref69] Song H. W. Zou Z. L. Kou J. Liu Y. Yang L. Z. Zilverstand A.. (2015). Love-related changes in the brain: a resting-state functional magnetic resonance imaging study. Front. Hum. Neurosci. 9:13. doi: 10.3389/fnhum.2015.00071, PMID: 25762915 PMC4327739

[ref70] Sorokowski P. Sorokowska A. Karwowski M. Groyecka A. Aavik T. Akello G.. (2021). Universality of the triangular theory of love: adaptation and psychometric properties of the triangular love scale in 25 countries. J. Sex Res. 58, 106–115. doi: 10.1080/00224499.2020.1787318, PMID: 32783568

[ref71] Sorokowski P. Zelazniewicz A. Nowak J. Groyecka A. Kaleta M. Lech W.. (2019). Romantic love and reproductive hormones in women. Int. J. Environ. Res. Public Health 16:4224. doi: 10.3390/ijerph16214224, PMID: 31683520 PMC6861983

[ref72] Sprecher S. Aron A. Hatfield E. Cortese A. Potapova E. Levitskaya A. (1994). Love: American style, Russian style, and Japanese style. Pers. Relat. 1, 349–369. doi: 10.1111/j.1475-6811.1994.tb00070.x

[ref73] Sternberg R. J. (1986). A triangular theory of love. Psychol. Rev. 93, 119–135. doi: 10.1037/0033-295x.93.2.119

[ref74] Sternberg R. J. (1988). The triangle of love: intimacy, passion, commitment. New York, NY: Basic Books.

[ref75] Sternberg R. J. (1997). Construct validation of a triangular love scale. Eur. J. Soc. Psychol. 27, 313–335. doi: 10.1002/(sici)1099-0992(199705)27:3<313::Aid-ejsp824>3.3.Co;2-w

[ref76] Stinson D. A. Cameron J. J. Hoplock L. B. (2021). The friends-to-lovers pathway to romance: prevalent, preferred, and overlooked by science. Soc. Psychol. Personal. Sci. 13, 562–571. doi: 10.1177/19485506211026992, PMID: 35251491 PMC8892041

[ref77] Stoessel C. Stiller J. Bleich S. Boensch D. Doerfler A. Garcia M.. (2011). Differences and similarities on neuronal activities of people being happily and unhappily in love: a functional magnetic resonance imaging study. Neuropsychobiology 64, 52–60. doi: 10.1159/000325076, PMID: 21606659

[ref78] Takahashi K. Mizuno K. Sasaki A. T. Wada Y. Tanaka M. Ishii A.. (2015). Imaging the passionate stage of romantic love by dopamine dynamics. Front. Hum. Neurosci. 9:6. doi: 10.3389/fnhum.2015.00191, PMID: 25914637 PMC4391262

[ref79] Tennov D. (1979). Love and limerence: the experience of being in love. New York, NY: Stein & Day.

[ref80] Tripartite Meeting of Experts on Labour Statistics on Updating the International Standard Classification of Occupations. (2007). Resolution concerning updating the international standard classification of occupations. Geneva: United Nations.

[ref81] Wang A. Y. Nguyen H. T. (1995). Passionate love and anxiety - a cross-generational study. J. Soc. Psychol. 135, 459–470. doi: 10.1080/00224545.1995.9712215, PMID: 7564306

[ref82] Wang C. Song S. S. Uquillas F. D. Zilverstand A. Song H. W. Chen H.. (2020). Altered brain network organization in romantic love as measured with resting-state fMRI and graph theory. Brain Imaging Behav. 14, 2771–2784. doi: 10.1007/s11682-019-00226-0, PMID: 31898089

[ref83] Wang Y. Zhang Y. T. Chen Y. Jing F. Wang Z. N. Hao Y. R.. (2016). Modulatory effect of romantic love on value estimation and its neural mechanism. Neuroreport 27, 323–328. doi: 10.1097/wnr.0000000000000541, PMID: 26854902

[ref84] Weisman O. Schneiderman I. Zagoory-Sharon O. Feldman R. (2015). Early stage romantic love is associated with reduced daily cortisol production. Adapt. Hum. Behav. Physiol. 1, 41–53. doi: 10.1007/s40750-014-0007-z

[ref85] Xu X. M. Aron A. Brown L. Cao G. K. Feng T. Y. Weng X. C. (2011). Reward and motivation systems: a brain mapping study of early-stage intense romantic love in Chinese participants. Hum. Brain Mapp. 32, 249–257. doi: 10.1002/hbm.21017, PMID: 21229613 PMC6870433

[ref86] Xu X. M. Brown L. Aron A. Cao G. K. Feng T. Y. Acevedo B.. (2012a). Regional brain activity during early-stage intense romantic love predicted relationship outcomes after 40 months: an fMRI assessment. Neurosci. Lett. 526, 33–38. doi: 10.1016/j.neulet.2012.08.004, PMID: 22902992

[ref87] Xu X. M. Wang J. Aron A. Lei W. Westmaas J. L. Weng X. C. (2012b). Intense passionate love attenuates cigarette Cue-reactivity in nicotine-deprived smokers: an fMRI study. PLoS One 7:e42235. doi: 10.1371/journal.pone.0042235, PMID: 22860092 PMC3409150

[ref88] Yin J. Zhang J. X. Xie J. Zou Z. L. Huang X. T. (2013). Gender differences in perception of romance in Chinese college students. PLoS One 8:e76294. doi: 10.1371/journal.pone.0076294, PMID: 24146853 PMC3797815

[ref89] Yin J. Zou Z. L. Song H. W. Zhang Z. Yang B. Huang X. T. (2018). Cognition, emotion and reward networks associated with sex differences for romantic appraisals. Sci. Rep. 8:2835. doi: 10.1038/s41598-018-21079-5, PMID: 29434208 PMC5809561

[ref90] Younger J. Aron A. Parke S. Chatterjee N. Mackey S. (2010). Viewing pictures of a romantic partner reduces experimental pain: involvement of neural reward systems. PLoS One 5:e13309. doi: 10.1371/journal.pone.0013309, PMID: 20967200 PMC2954158

[ref91] Zeki S. Romaya J. P. (2010). The brain reaction to viewing faces of opposite- and same-sex romantic partners. PLoS One 5:e15802. doi: 10.1371/journal.pone.0015802, PMID: 21209829 PMC3013131

