# Supplementary material for: Toward consistent reporting of sample characteristics in studies investigating the biological mechanisms of romantic love
Source: Front Psychol. 2023 May 4;14:983419. doi: 10.3389/fpsyg.2023.983419 (PMC10192910; doi:10.3389/fpsyg.2023.983419)
Supplement: Supplementary file 1 [file Table_1.docx]

| **Supplementary Table 1.** **Romantic love sample characteristics reported in neuroimaging studies with a group or entire sample experiencing romantic love** | | | | | | |
| --- | --- | --- | --- | --- | --- | --- |
| **Reference** | **n** | **Female/ women n** | **Age** | **Measure of romantic love** | **Relationship duration/ time in love** | **Descriptors** |
| **EEG/ERP studies** | | | | | | |
| Langeslag, Jansma, Franken, Van Strien (2007) | 18 | 9 (50.0%) | 18-34 years (Mean=21.5) | Self-reported love (9-point scale)=8.4 (SD=0.6); PLS mean item score=7.8 (SD=0.5, range 7.0-8.5) | Mean duration of relationships=12.1 months (SD=9.4);  Time in love mean=12.6 months (SD=10.1, range 2.5-36) | Students; heterosexual; normal or corrected vision; healthy; no medication; right-handed |
| Langeslag, Franken, Van Strien (2008) | 20 | 11 (55.0%) | 18-23 years (Mean=19.3) | Self-reported love (9-point scale)=7.3 (SD=0.9); PLS mean item score=6.8 (SD=0.8) | Duration < 7 months; Mean duration of relationships=3.1 months (SD=1.7, range 0.5-6.5);  Time in love mean=3.7 months (SD=1.8, range 0.8-6.5) | Psychology students; heterosexual; normal or corrected vision; healthy; no medication affecting CNS; right-handed |
| Cacioppo, Grafton, Bianchi-Demicheli (2012) | 20 | 20 (100%) | High passionate love group n=10; age range18-22 years (Mean=19.1); | High passionate love PLS mean item score=8 (SD=0.73); | High passionate love participants’ time in love range=2-42 months; | Students from Dartmouth college, USA; English speakers; normal or corrected vision; healthy with no prior or current neurological or psychiatric disorders; not taking antidepressant medication; right-handed |
|  |  |  | Low passionate love group n=10; age range 18-26 years (Mean=20.4) | Low passionate love PLS mean item score=7.27 (SD=0.45) | Low passionate love participants’ time in love range=2-36 months |  |
| Langeslag, Olivier, Kὄhlen, Nijs, Van Strien (2015) | S1: 20 | 15 (75.0%) | 18-28 years (Mean=21.1) | PLS (Range mean item score=5.9-8.8; Mean item score=7.7) | Relationship length mean=6.1 months (range 1-12);  Time in love mean=6.9 months (range=1-11 months) | Erasmus University; Rotterdam, Netherlands; normal or corrected vision; no mental disorder; no medication affecting CNS; right-handed |
|  | S2: 18 | 15 (83.3%) | 18-26 years (Mean=20.9) | PLS (Range mean item score=4.6-8.3; Mean item score=6.8); IS (Range mean item score=1.4-5.1; Mean item score=3.2); AS (Range mean item score=3.3-7.0; Mean item score=5.7) | Relationship length mean=6.5 months (range 0.5-16);  Time in love mean =7.3 months (range=2-17.5) |  |
| Langeslag & Van Strien (2016) | S1: 32 | 25 (78.1%) | 18-30 years  (Mean=21.4) | IS range mean item score=1.5-5.6 (Mean=3.4); AS range mean item score=3.5-6.9 (Mean=5.8); PLS range mean item score=4.2-8.4 (Mean=7.2) | Relationship length mean=6.5 months (range 3-17);  Time in love mean=8.9 months (range=1-63) | University of Maryland, USA, heterosexual |
| Langeslag & Van Strien (2019) | 24 | 15 (62.5%) | 18-35 years (Mean=23.9) | Questionnaire; PLS (Range mean item score=4.1-9.0; Mean item score=7.1) | Time in love <1 year (Range=1-12 months; Mean=6.4); | St Louis, USA; heterosexual; normal or corrected vision; no neurological or mental disorders; no medication affecting CNS; right-handed |
| Langeslag & Van Strien (2020) | S1: 24 | 11 (45.8%) | 18-28 years (Mean=20.9) | IS range mean item score=1.8-5 (Mean=3.2); AS range mean item score=4.2-6.9 (Mean=5.8); IAS Obsession range mean item score=2.3-5.3 (Mean=3.7); PLS range mean item score=5.1-8.2 (Mean=7.0); PLS Obsession range mean item score=3-7.3 (Mean=5.7) | Relationship length mean=5.3 months (range 0.3-15);  Time in love mean=6.4 months (range 1-12) | Students from Erasmus University Rotterdam, Netherlands; heterosexual; normal or corrected vision; no neurological or mental disorders; no medication affecting CNS; right-handed |
|  | S2: 24 | 15  (62.5%) | 18-34 years (Mean=22.4) | IS range mean item score=2.3-6.2 (Mean=3.8); AS Attachment range; Mean item score=1.1-6.9 (Mean=5.6); IAS Obsession range mean item score=1.7-6.7 (Mean=4.3); PLS range mean item score=4.9-8.8 (Mean=7.5); PLS Obsession range mean item score=3-9 (Mean=6.7) | Relationship length mean=5.8 months (range 1-10);  Time in love mean=6.3 months (range 1.8-12) | Students from University of Missouri – St Louis, USA; heterosexual; normal or corrected vision; no neurological or mental disorders; no medication affecting CNS; right-handed |
| Cannas Aghedu, Sarlo, Zappasodi, Acevedo, & Bisiacchi (2021) | 22 | 19  (86.4%) | 20-29 years (Mean=23.09 [SD=2.59]) | Self-report (10-point scale); PLS (Mean=99.86 [SD=7.97]) | Relationship length mean=30 months (range 5-54 [SD=18]) | Students from University of Padova, Italy; normal or corrected vision; no neurological or psychiatric conditions; |
| **fMRI studies** | | | | | | |
| Bartels & Zeki (2000) | 17 | 11  (64.7%) | 21-37 years (Mean=24.5) | Short written statement; PLS (Mean item score=7.55 [SD=0.97]) | Relationship length mean=28.8 months (SD=20.4) | right-handed except for one male subject; eleven countries and several ethnic groups were represented |
| Aron et al. (2005) | 17 | 10  (58.8%) | 18-26 years (Mean=20.6) | Self-report questionnaire; PLS (Mean item score=8.54 | Time in love mean=7.4 months (range 1-17) | State University of New York at Stony Brook community, the Rutgers University community, and the New York City area, USA; not taking antidepressants; right-handed |
| Ortigue, Bianchi-Demicheli, Hamilton, & Grafton (2007) | 36 | 36  (100%) | Mean=20.1 years (SD=3.2) | Semi-structured interview; Intensity of love; Percentage of time thinking about beloved in waking hours; PLS (Mean item score=7.7 [SD=1.22)] | Time in love mean=15.3 months (range 1-60 months [SD=14.5]) | Dartmouth College, USA; heterosexual; dating, engaged, or married; had a favourite passion in life; normal or corrected vision; not taking antidepressants; no chemical dependencies; no neurological or psychiatric disorders; right-handed |
| Kim et al. (2009) | 10 | 5 (50.0%) | 18-24 years (Mean=21.1 [SD=1.97]) | PLS (Baseline mean f=120.2 [SD=7]; Baseline mean m=118.6 [SD=9.1]; Follow-up mean f=106.2; [SD=3.0];  Follow-up mean m=110.8 [SD=4.0]) | Time in love ≤100 days at baseline | Heterosexual couples; psychologically, medically and neurologically healthy; right-handed |
| Fisher, Brown, Aron, Strong, & Mashek (2010) | 15 | 10 (66.6%) | 18-21 years (Mean=19.8 [SD=1.0]) | PLS (Mean item score=8.0 [SD=0.6]) | Relationship length mean=21 months (range 4-48);  Time since breakup 1-32 weeks (mean=63 days) | State University of New York at Stony Brook, Rutgers University, and the New York area, USA; heterosexual; not taking antidepressants; right-handed; |
| Younger, Aron, Parke, Chatterjee, & Mackey (2010) | 15 | 8 (53.3%) | 19-21 years (Mean=20) | 15-item PLS (Range=91.5-132; Mean=109.8 [SD=11.2]) | Time in love ≤ 9 months | Right-handed; students |
| Zeki & Romaya (2010) | 24^a^ | 12 (50.0%) | 19-47 years (Mean=26.3 [SD=6.4]) | PLS (Range=61-117; Mean=100.1) | Relationship length range  4 months-23 years (Mean=3.7 years [SD=4.4]) | Equally divided between sexes and sexuality; Mostly right-handed; West European, East European, American, Oriental and Asian backgrounds |
| Xu et al., (2011) | 18 | 10 (55.5%) | 19-25 years (Mean21.61 [SD=1.75]) | - | Relationship length range 1.3-13 months (Mean=6.54 [SD=3.19]) | Chinese; students; right-handed; no psychotropic medication |
| Stoessel et al. (2011) | 12 | 6 (50.0%) | HL: Mean=24.2 years (SD=4.02) | HL: PLS (Mean f=112.4 [SD=14.76]; Mean m=109.8 [12.69]) | HL Time in love ≤ 6 months; | Right-handed; heterosexual; physically and neurologically healthy with no previous history of substance abuse, psychotropic medication intake, psychotic episodes, or affective disorders; not pregnant or lactating; no fMRI contraindications |
|  |  |  | UL: Mean=24.08 years (SD=4.5) |  | UL: Time after separation ≤ 6 months |  |
| Acevedo, Aron, Fisher, & Brown (2012) | 17 | 10 (58.8%) | 39-67 years (Mean=52.85 [SD=8.91]) | PLS (Mean item score=5.51 [SD=0.36]); Eros-LAS (Mean item score=5.76 [SD=0.26]) | Married for 10-29 years (Mean= 21.4, [SD = 5.89]) with  0-4 children (Mean=1.9);  Known their partner for 24.18 years (SD=6.42) | Healthy, right-handed, heterosexuals, married; avg 16 years of education, 76% Caucasian, 12 Asian-American, 12% Latino/a; recruited in NY metro area; Mean weekly sexual activity=2.2 (SD=1.85); not taking antidepressants; no fMRI contraindications |
| Langeslag, Van der Veen, & Röder (2014) | 15 | 9 (60.0%) | 18-25 years (Mean=20.8) | Self-report intensity on 9 point scale (Range=6-9 Mean=7.9 [SD=1.0]); PLS (Range mean item score=5.1-8.8; Mean item score=7.2 [SD=0.9]) | Time in love range=2.5-8 months (Mean=5.1; SD=1.6) | University students, Erasmus University Rotterdam; heterosexual; normal or corrected vision; no medical diagnosis; no medication affecting CNS; no fMRI contraindications; right-handed |
| Xu et al. (2012a) | 18 | 0 (0.0%) | 21-33 years (Mean=25.11 [SD=3.03]) | 14-item PLS (Mean item score=7.75 [SD=0.82]) | Relationship length mean=14.22 months (SD=10.97) | Chinese; students; smoker for at least 6 months; mostly right-handed; no psychoactive medication or severe alcohol/drug use; no history of claustrophobia or head trauma; no fMRI contraindications |
| Xu et al. (2012b) | 12 | 7 (58.3%) | Mean=21.5 years (SD=1.62) | - | Relationship length mean at the time of scan=5.78 months (SD=2.53) | Chinese; students; right-handed; no psychoactive medications; 6 out of 12 follow-up participants were still in a relationship with their partner 40 months after scan |
| Scheele et al. (2013) | DSC: 20 | DSC: 0 (0%) | DSC: Mean=25.1 years (SD=3.3) | DSC: PLS mean item score for experimental condition=6.33 (SD=0.96); Mean item score for placebo condition=6.46 (SD=1.21); MEIL Mean “Eros” score=7.19 (SD=0.99) | DSC: Relationship length mean=28.8 months (SD=15.4) | Heterosexual; pair-bonded; unmarried and no children; non-smoking; healthy; no current or past physical or psychiatric illness; naive to prescription-strength psychoactive medication; had not taken any over-the-counter psychoactive medication in the last 4 weeks |
|  | RPL: 20 | RPL: 0 (0%) | RPL: Mean=26.6 (SD=3.8) | RPL: PLS mean item score for experimental condition=6.46 (SD=1.36); Mean item score for placebo condition=6.49 (SD=1.27); MEIL Mean “Eros” score=6.98 (SD=1.55) | RPL: Relationship length mean=36.4 months (SD=25.3) |  |
| Yin, Zhang, Xie, Zou, & Huang (2013) | 36 | 18 (50.0%) | - | PLS>4.8 (Mean=5.20 [SD=0.27]) | Relationship length range 4-48 months (Mean=18.42 [SD=10.65]) | Right-handed, heterosexual; no neurological or psychiatric disorders; |
| Song et al. (2015) | 34 | - | ≥18 years | PLS (Mean=104.21 [10.58]) | Time in love (Range=4-18 months; Mean=12.21 [SD=3.33]) | Students; Southwestern University, China; healthy; heterosexual |
| Wang et al. (2016) | 22 | 11 (50.0%) | Mean=22.51 years (SD=2.19) | Self-report (10-point scale) (Range=6-10; Mean=8.19 [SD=1.52]) | Time in love range 3 months – 2 years | Graduate or undergraduate students; |
| Yin et al., (2018) | 32^4^ | 16 (50.0%) | Females: Mean=20.81 years (SD=2.27); Males: Mean=21.19 (SD=2.29) | PLS (Mean item score=4.43 [SD=0.61]) | 3-18 months (Mean=8.76 [SD=5.42]) | Right-handed, Southwest University, mean communications time with partner was 3.98 hours per day, no neurological or psychiatric disorders, heterosexual |
| Acevedo, Poulin, Collins, & Brown (2020) | T1: 19  T2: 13 | 11 (57.9%)  7 (53.8%) | 21-32 years (T1 Mean age=27.21 [SD=3.29]) | Eros subscale of LAS (T1 mean item score=6.33 [SD=0.32]); (Mean T2 item score=6.17 [SD=0.87) | Relationship length mean T1=4.11 years [SD=3.09]) | First-time marriage; no children; good health; no claustrophobia; no pregnancy; no history of head trauma; no fMRI contraindications; right-handed, not pregnant; mostly college-educated; sexual frequency at baseline=2.95 times per week, at follow-up=1.83 |
| Wang et al. (2020) | 34 | 16 (47.1%) | Mean=21.21 (SD=2.42) | PLS (Mean=104.21 [SD=10.58]) | Time in love (Range=8-17 months; Mean=12.23 [SD=2.95]) | In a relationship with loved one; Education years avg=13.24; Students; Southwestern university, China. |
| **PET studies** | | | | | | |
| Takahashi et al. (2015) | 10 | 6 (60.0%) | Mean=27.4 years (SD=4.3) | PLS (Mean=201.9 [SD=33.7]) | Relationship length range 2-125 months (Median=17) | Heterosexual; normal or corrected vision; right-handed; no history of medical illness |
| ^a^= 4 subject were excluded and characteristics represent 28 original participants; ^b^= 10 subjects were excluded and characteristics represent 42 original participants; HL=happily in love group; UL= unhappily in love group; DSC=discovery study; RPL=replication study; S1=Study 1; S2=Study 2; PLS=Passionate Love Scale; LAS= Love Attitudes Scale; IS=Infatuation Scale; AS=Attachment Scale; IAS=Infatuation and Attachment Scale | | | | | | |

**References**

Acevedo, B. P., Aron, A., Fisher, H. E., & Brown, L. L. (2012). Neural correlates of long-term intense romantic love. *Social Cognitive and Affective Neuroscience, 7*(2), 145-159. doi:10.1093/scan/nsq092

Acevedo, B. P., Poulin, M. J., Collins, N. L., & Brown, L. L. (2020). After the Honeymoon: Neural and Genetic Correlates of Romantic Love in Newlywed Marriages. *Frontiers in Psychology, 11*(634). doi:10.3389/fpsyg.2020.00634

Aron, A., Fisher, H., Mashek, D. J., Strong, G., Li, H. F., & Brown, L. L. (2005). Reward, motivation, and emotion systems associated with early-stage intense romantic love. *Journal of Neurophysiology, 94*(1), 327-337. doi:10.1152/jn.00838.2004

Bartels, A., & Zeki, S. (2000). The neural basis of romantic love. *Neuroreport, 11*(17), 3829-3834. doi:10.1097/00001756-200011270-00046

Cacioppo, S., Grafton, S. T., & Bianchi-Demicheli, F. (2012). The Speed of Passionate Love, As a Subliminal Prime: A High-Density Electrical Neuroimaging Study. *Neuroquantology, 10*(4), 715-724. Retrieved from <Go to ISI>://WOS:000312210700013

Cannas Aghedu, F., Sarlo, M., Zappasodi, F., Acevedo, B. P., & Bisiacchi, P. S. (2021). Romantic love affects emotional processing of love-unrelated stimuli: An EEG/ERP study using a love induction task. *Brain and Cognition, 151*, 105733. doi:<https://doi.org/10.1016/j.bandc.2021.105733>

Fisher, H. E., Brown, L. L., Aron, A., Strong, G., & Mashek, D. (2010). Reward, Addiction, and Emotion Regulation Systems Associated With Rejection in Love. *Journal of Neurophysiology, 104*(1), 51-60. doi:10.1152/jn.00784.2009

Kim, W., Kim, S., Jeong, J., Lee, K. U., Ahn, K. J., Chung, Y. A., . . . Chae, J. H. (2009). Temporal Changes in Functional Magnetic Resonance Imaging Activation of Heterosexual Couples for Visual Stimuli of Loved Partners. *Psychiatry Investigation, 6*(1), 19-25. doi:10.4306/pi.2009.6.1.19

Langeslag, S., Olivier, J., Köhlen, M., Nijs, I., & Van Strien, J. (2015). Increased attention and memory for beloved-related information during infatuation: Behavioral and electrophysiological data. *Social Cognitive and Affective Neuroscience, 10*. doi:10.1093/scan/nsu034

Langeslag, S., & Steenbergen, H. (2019). Cognitive control in romantic love: the roles of infatuation and attachment in interference and adaptive cognitive control. *Cognition and Emotion, 34*, 1-8. doi:10.1080/02699931.2019.1627291

Langeslag, S. J., Jansma, B. M., Franken, I. H., & Van Strien, J. W. (2007). Event-related potential responses to love-related facial stimuli. *Biol Psychol, 76*(1-2), 109-115. doi:10.1016/j.biopsycho.2007.06.007

Langeslag, S. J., van der Veen, F. M., & Röder, C. H. (2014). Attention modulates the dorsal striatum response to love stimuli. *Hum Brain Mapp, 35*(2), 503-512. doi:10.1002/hbm.22197

Langeslag, S. J. E., Franken, I. H. A., & Van Strien, J. W. (2008). Dissociating love-related attention from task-related attention: An event-related potential oddball study. *Neuroscience Letters, 431*(3), 236-240. doi:<https://doi.org/10.1016/j.neulet.2007.11.044>

Langeslag, S. J. E., & van Strien, J. W. (2016). Regulation of Romantic Love Feelings: Preconceptions, Strategies, and Feasibility. *Plos One, 11*(8), e0161087. doi:10.1371/journal.pone.0161087

Langeslag, S. J. E., & van Strien, J. W. (2020). Preferential processing of task-irrelevant beloved-related information and task performance: Two event-related potential studies. *Neuropsychologia, 145*, 106497. doi:10.1016/j.neuropsychologia.2017.09.015

Ortigue, S., Bianchi-Demicheli, F., Hamilton, A., & Grafton, S. T. (2007). The neural basis of love as a subliminal prime: An event-related functional magnetic resonance imaging study. *Journal of Cognitive Neuroscience, 19*(7), 1218-1230. doi:10.1162/jocn.2007.19.7.1218

Scheele, D., Wille, A., Kendrick, K. M., Stoffel-Wagner, B., Becker, B., Gunturkun, O., . . . Hurlemann, R. (2013). Oxytocin enhances brain reward system responses in men viewing the face of their female partner. *Proceedings of the National Academy of Sciences of the United States of America, 110*(50), 20308-20313. doi:10.1073/pnas.1314190110

Song, H. W., Zou, Z. L., Kou, J., Liu, Y., Yang, L. Z., Zilverstand, A., . . . Zhang, X. C. (2015). Love-related changes in the brain: a resting-state functional magnetic resonance imaging study. *Frontiers in Human Neuroscience, 9*, 13. doi:10.3389/fnhum.2015.00071

Stoessel, C., Stiller, J., Bleich, S., Boensch, D., Doerfler, A., Garcia, M., . . . Forster, C. (2011). Differences and Similarities on Neuronal Activities of People Being Happily and Unhappily in Love: A Functional Magnetic Resonance Imaging Study. *Neuropsychobiology, 64*(1), 52-60. doi:10.1159/000325076

Takahashi, K., Mizuno, K., Sasaki, A. T., Wada, Y., Tanaka, M., Ishii, A., . . . Watanabe, Y. (2015). Imaging the passionate stage of romantic love by dopamine dynamics. *Frontiers in Human Neuroscience, 9*, 6. doi:10.3389/fnhum.2015.00191

Wang, C., Song, S. S., Uquillas, F. D., Zilverstand, A., Song, H. W., Chen, H., & Zou, Z. L. (2020). Altered brain network organization in romantic love as measured with resting-state fMRI and graph theory. *Brain Imaging and Behavior*. doi:10.1007/s11682-019-00226-0

Wang, Y., Zhang, Y. T., Chen, Y., Jing, F., Wang, Z. N., Hao, Y. R., . . . Zhang, X. C. (2016). Modulatory effect of romantic love on value estimation and its neural mechanism. *Neuroreport, 27*(5), 323-328. doi:10.1097/wnr.0000000000000541

Xu, X. M., Aron, A., Brown, L., Cao, G. K., Feng, T. Y., & Weng, X. C. (2011). Reward and Motivation Systems: A Brain Mapping Study of Early-Stage Intense Romantic Love in Chinese Participants. *Human Brain Mapping, 32*(2), 249-257. doi:10.1002/hbm.21017

Xu, X. M., Brown, L., Aron, A., Cao, G. K., Feng, T. Y., Acevedo, B., & Weng, X. C. (2012). Regional brain activity during early-stage intense romantic love predicted relationship outcomes after 40 months: An fMRI assessment. *Neuroscience Letters, 526*(1), 33-38. doi:10.1016/j.neulet.2012.08.004

Xu, X. M., Wang, J., Aron, A., Lei, W., Westmaas, J. L., & Weng, X. C. (2012). Intense Passionate Love Attenuates Cigarette Cue-Reactivity in Nicotine-Deprived Smokers: An fMRI Study. *Plos One, 7*(7), 9. doi:10.1371/journal.pone.0042235

Yin, J., Zhang, J. X., Xie, J., Zou, Z. L., & Huang, X. T. (2013). Gender Differences in Perception of Romance in Chinese College Students. *Plos One, 8*(10). doi:10.1371/journal.pone.0076294

Yin, J., Zou, Z. L., Song, H. W., Zhang, Z., Yang, B., & Huang, X. T. (2018). Cognition, emotion and reward networks associated with sex differences for romantic appraisals. *Scientific Reports, 8*, 11. doi:10.1038/s41598-018-21079-5

Younger, J., Aron, A., Parke, S., Chatterjee, N., & Mackey, S. (2010). Viewing Pictures of a Romantic Partner Reduces Experimental Pain: Involvement of Neural Reward Systems. *Plos One, 5*(10). doi:10.1371/journal.pone.0013309

Zeki, S., & Romaya, J. P. (2010). The Brain Reaction to Viewing Faces of Opposite- and Same-Sex Romantic Partners. *Plos One, 5*(12). doi:10.1371/journal.pone.0015802
