# Supplementary material for: Toward consistent reporting of sample characteristics in studies investigating the biological mechanisms of romantic love
Source: Front Psychol. 2023 May 4;14:983419. doi: 10.3389/fpsyg.2023.983419 (PMC10192910; doi:10.3389/fpsyg.2023.983419)
Supplement: Supplementary file 2 [file Table_2.docx]

| **Supplementary Table 2. Romantic love sample characteristics reported in endocrinological studies with a group or entire sample experiencing romantic love** | | | | | | |
| --- | --- | --- | --- | --- | --- | --- |
| **Reference** | **n** | **Female/ woman n** | **Age** | **Measure of romantic love** | **Relationship duration/ time in love** | **Descriptors** |
| Marazziti, Akiskal, Rossi, & Cassano (1999) | 20 | 17 (85.0%) | Mean=24 years (SD=3) | ≥ 4 hours per day thinking about loved one | Relationship length ≤6 months | No sexual intercourse; medical students; healthy |
| Marazziti & Canale (2004) | 24 | 12 (50.0%) | Mean=27 years (SD=4) | ≥ 4 hours per day thinking about loved one (Mean=9 [SD=3]) | Relationship length ≤6 months (Mean=3 [SD=1]) | Medical residents and students, most had regular sexual activity; no contraceptive pills; the men had no history of genital disease or hypogonadism;  healthy; no personal or family history of major psychiatric disorders; never taken psychotropic drugs, apart from three who occasionally took benzodiazepines for sleep; not heavy smokers; did not belong to high-risk HIV group |
| Emanuele et al. (2006) | 58 | 37 (63.8%) | 18-31 years (Mean=24.4 [SD=3.8]) | ≥ 4 hours per day thinking about loved one; 15-item PLS >85 | Relationship length ≤6 months | Italian, healthy; not heavy smoker; not HIV positive; no first-degree relatives with psychiatric disorder; no psychotropic medication |
| Dundon & Rellini (2012) | 29 | 29 (100%) | - | 3-item of PLS ≥17 (out of 27); 30-item PLS (results not reported) | Relationship length ≤6 months | Students; mostly Caucasian; majority (89%) identified as exclusively or predominantly heterosexual; had sexual activity within past month; not pregnant or lactating; no psychiatric admission in past year; never experienced sexual abuse; not experiencing depression or sexual dysfunction; not taking antidepressants, antipsychotic medications, or beta-blockers |
| Langeslag, van der Veen, & Fekkes (2012) | 20 | 10 (50.0%) | Females: Mean=19.7 years (SD=2.3); Males: Mean=21.3 (SD=2.7) | Self-reported love intensity range 5-9 (Mean=7.9 [SD1.9]); PLS range 3.6-8.1 (Mean=6.8 [SD=1]); Percentage of waking hours spent thinking about loved one (Mean=65%) | Relationship length range 1.8-8.5 months (Mean=5.1 [SD=1.9);  Time in love (Range=1-9 months; Mean=4.6 [SD=2.4]) | Students of the Erasmus University Rotterdam; mostly heterosexual; no medical history of depression, OCD, hypercholesterolemia, or hypertension; not taking antidepressants, statins, or antihypertensives; |
| Weisman, Schneiderman, Zagoory-Sharon, & Feldman (2015) | 120 | 60 (50.0%) | Mean=23.93 (SD=6.6) | TLS (Passion mean=107; Intimacy mean=116; Commitment mean=116) | Relationship length range 2 weeks-4 months (Mean=2.4 months) | Non-cohabitating heterosexual adults; mostly students; middle-class background; ages of less than 35 years old; completed minimum of high-school education; not taking any medication; generally healthy; |
| Marazziti et al. (2017) | 30 | 15 (50.0%) | 20-40 years (Mean=29.8 ([SD=4]) | 14-item self-report instrument developed by the research group | Relationship length ≤6 months (Mean=3 [SD=1]) | Healthy; mostly heterosexual; no personal or family history of major psychiatric disorders; |
| Sorokowski et al. (2019) | 47 | 47 (100%) | 25-30 years | TLS (Passion subscale) | Relationship length range  3-6 months | Healthy; no fertility problems; no recent antibiotic or anti-inflammatory use; no evidence of ongoing infection; no contraception or hormone treatment; normal range of hormone levels; normal BMI; no children, heterosexual |
| Renner, Stanulla, Walther, & Schindler (2021) | I:16 | I:16 (100%) | I:18-29 years  Mean=21.44 (SD=2.28) | Subjective degree of infatuation (0-100) (I: Mean=82.94 [SD=15.73]; PLS (I: Mean=200.94 [SD=39.21] | I: Mean=2.46 (SD=1.33) | Students; identified as cis woman; hair length ≥1 cm; no chronic physical or psychological illnesses; no endocrine medication use within preceding 6 weeks; not smoking >15 cigarettes a day; not pregnant or lactating; not a partner of another study participant |
|  | LT:40 | LT:40 (100%) | LT:18-29 years  Mean=20.38 (SD=2.08) | Subjective degree of infatuation LT: Mean=79.05 [SD16.62]);  PLS LT: Mean=214.73 [SD=22.42]) | LT: Mean=35.27 (SD=19.06) |  |
| I=infatuation group; LT=long-term relationship group; PLS=Passionate Love Scale; TLS=Triangular Love Scale; MEIL=Marburg Attitude Scales towards Love Styles | | | | | | |

**References**

Dundon, C. M., & Rellini, A. H. (2012). Emotional States of Love Moderate the Association Between Catecholamines and Female Sexual Responses in the Laboratory. *Journal of Sexual Medicine, 9*(10), 2617-2630. doi:10.1111/j.1743-6109.2012.02799.x

Emanuele, E., Politi, P., Bianchi, M., Minoretti, P., Bertona, M., & Geroldi, D. (2006). Raised plasma nerve growth factor levels associated with early-stage romantic love. *Psychoneuroendocrinology, 31*(3), 288-294. doi:10.1016/j.psyneuen.2005.09.002

Langeslag, S. J. E., van der Veen, F. M., & Fekkes, D. (2012). Blood Levels of Serotonin Are Differentially Affected by Romantic Love in Men and Women. *Journal of Psychophysiology, 26*(2), 92-98. doi:10.1027/0269-8803/a000071

Marazziti, D., Akiskal, H. S., Rossi, A., & Cassano, G. B. (1999). Alteration of the platelet serotonin transporter in romantic love. *Psychological Medicine, 29*(3), 741-745. doi:10.1017/s0033291798007946

Marazziti, D., Baroni, S., Giannaccini, G., Piccinni, A., Mucci, F., Catena-Dell'Osso, M., . . . Dell'Osso, L. (2017). Decreased lymphocyte dopamine transporter in romantic lovers. *Cns Spectrums, 22*(3), 290-294. doi:10.1017/s109285291600050x

Marazziti, D., & Canale, D. (2004). Hormonal changes when falling in love. *Psychoneuroendocrinology, 29*(7), 931-936. doi:10.1016/j.psyneuen.2003.08.006

Renner, J., Stanulla, M., Walther, A., & Schindler, L. (2021). CortiLove: A pilot study on hair steroids in the context of being in love and separation. *Psychoneuroendocrinology, (Comprehensive Psychoneuroendocrinology)*. doi:10.1016/j.cpnec.2021.100061

Sorokowski, P., Zelazniewicz, A., Nowak, J., Groyecka, A., Kaleta, M., Lech, W., . . . Pisanski, K. (2019). Romantic Love and Reproductive Hormones in Women. *International Journal of Environmental Research and Public Health, 16*(21). doi:10.3390/ijerph16214224

Weisman, O., Schneiderman, I., Zagoory-Sharon, O., & Feldman, R. (2015). Early Stage Romantic Love is Associated with Reduced Daily Cortisol Production. *Adaptive Human Behavior and Physiology, 1*(1), 41-53. doi:10.1007/s40750-014-0007-z
