# Supplementary material for: Toward consistent reporting of sample characteristics in studies investigating the biological mechanisms of romantic love
Source: Front Psychol. 2023 May 4;14:983419. doi: 10.3389/fpsyg.2023.983419 (PMC10192910; doi:10.3389/fpsyg.2023.983419)
Supplement: Supplementary file 3 [file Table_3.docx]

| **Supplementary Table 3. Romantic love sample characteristics reported in genetics studies with a group or entire sample experiencing romantic love** | | | | | | |
| --- | --- | --- | --- | --- | --- | --- |
| **Reference** | **n** | **Female/ woman n** | **Age** | **Measure of romantic love** | **Relationship duration/ time in love** | **Descriptors** |
| Murray, Haselton, Fales, & Cole (2019) | 17 | 17  (100%) | Mean age 20.5 years (SD=2.6) ^a^ | Self-report | <1 month at baseline; Median days since enrolment=92 days (SD=112) | American; Student; heterosexual; not in love at baseline; not pregnant or breastfeeding; non-smoker; not taking immune, cardiovascular, or psychiatric medication; ethnicities: 36.2% White or Caucasian, 25.5% Asian, 17.0% multi-racial, 12.8% Hispanic or Latino, 6.4% Black or African American, 2.1% American Indian; BMI mean=22.1 (SD=3.6) |
| Acevedo, Poulin, Collins, & Brown (2020) | T1: 19 | 11 (57.9%) | 21-32 years (T1 Mean age=27.21 [SD=3.29]) | Eros subscale of LAS (T1 mean item score=6.33 [SD=0.32]); | Relationship length mean T1=4.11 years [SD=3.09] | First-time marriage; no children; good health; no claustrophobia; no pregnancy; no history of head trauma; no fMRI contraindications; right-handed, not pregnant; mostly college-educated; sexual frequency at baseline=2.95 times per week, at follow-up=1.83 |
|  | T2: 13 | T2:7 (53.8%) |  | T2 item mean score=6.17 [SD=0.87] |  |  |
| ^a^= Mean age for all participants, not just love group; T1=Baseline; T2=Follow-up | | | | | | |

**References**

Acevedo, B. P., Poulin, M. J., Collins, N. L., & Brown, L. L. (2020). After the Honeymoon: Neural and Genetic Correlates of Romantic Love in Newlywed Marriages. *Frontiers in Psychology, 11*(634). doi:10.3389/fpsyg.2020.00634

Murray, D. R., Haselton, M. G., Fales, M., & Cole, S. W. (2019). Falling in love is associated with immune system gene regulation. *Psychoneuroendocrinology, 100*, 120-126. doi:10.1016/j.psyneuen.2018.09.043
